# Experimental Study on the Effect of Limestone Powder Content on the Dynamic and Static Mechanical Properties of Seawater Coral Aggregate Concrete (SCAC)

**DOI:** 10.3390/ma16093381

**Published:** 2023-04-26

**Authors:** Juan Qi, Lili Jiang, Ming Zhu, Chaomin Mu, Rui Li

**Affiliations:** 1 School of Electrical and Information Engineering, Anhui University of Science and Technology, Huainan 232001, China; 2 School of Civil Engineering and Architecture, Anhui University of Science and Technology, Huainan 232001, China; 3 School of Safety Science and Engineering, Anhui University of Science and Technology, Huainan 232001, China

**Keywords:** SCAC, SHPB, mechanical properties, limestone powder

## Abstract

The development of island construction concrete can serve as a basis for the development and utilization of island resources. Complying with the principle of using local materials to configure seawater coral aggregate concrete (SCAC) that is able to meet the requirements of island and reef engineering construction could effectively shorten the construction period and cost of island and reef engineering construction. In this paper, quasi-static mechanical experiments and dynamic mechanical experiments were carried out on SCAC with different limestone powder contents. High-speed photography technology and Digital Image Correlation (DIC) were used to monitor the dynamic failure process and strain field of SCAC, and the influence of limestone powder content on the dynamic and static mechanical properties of SCAC was investigated. The results showed that, when the limestone powder content was 20% and 16%, the quasi-static compressive strength and quasi-static tensile strength exhibited the best improvement. Additionally, with increasing limestone powder content, the dynamic tensile strength of SCAC first showed and increasing trend and then a decreasing trend, reaching its maximum value when the limestone powder content was 16%. Moreover, the maximum strain value of SCAC with the same limestone powder content increased with increasing strain rate grade, showing an obvious effect on strain rate.

## 1. Introduction

The design and construction of island engineering projects such as island airports, island buildings, and docks serve as a basis for the development of marine resources [1,2,3]. However, in the process of island construction, transportation costs and construction durations will undoubtedly be increased if all building materials (especially concrete) need to be transported by land [4,5,6]. In addition, due to the special environment of islands, island buildings and constructions inevitably face the threat of dynamic loading, resulting from phenomena such as earthquakes and explosions [7,8]. Therefore, methods for producing and processing seawater coral aggregate concrete (SCAC) enable to meet the requirements of island and reef engineering construction using local materials in order to reduce economic costs and shorten construction periods while satisfying the needs of island and reef engineering construction projects is a key issue in island and reef construction.

The research on SCAC can be traced back to World War II [9]. With the development of marine resources, the performance of SCAC is becoming a hot topic in research on the development of marine and island resources. Over nearly half a century, researchers have carried out research to differing extents on various aspects of SCAC performance (such as corrosion resistance, durability, mechanical properties, etc.), and some progress has been made [10]. Studies have shown that, due to the low strength, ease of crushing, high porosity and high permeability of coral aggregate particles, the strength of SCAC mixed with coral aggregate and seawater is not ideal [11]. The original coral concrete exhibited a relatively low compressive strength of approximately 30 MPa [10], which is not able to satisfy the requirements of island and reef engineering construction [12]. Therefore, current research on SCAC is focused on ways of improving the performance of SCAC, including its mechanical properties. Studies have shown that the mechanical properties of concrete are closely related to the composition and structure of concrete [13,14,15,16]. Some scholars have explored ways of improving the strength of SCAC by optimizing the mix ratio [8,17,18], while others have attempted to improve the performance of SCAC by adding fibers to SCAC. Some scholars have attempted the addition of plant fibers (such as sisal fibers) to SCAC to improve its performance [7,19,20]. Xu et al. [21] added glass fibers to SCAC and developed a method for analyzing the development of internal cracks in glass-fiber-reinforced polymer–sea sand concrete composites, and the strength enhancement effect of glass fibers on SCAC was studied. Liu et al. [22] studied the effect of the addition of carbon fibers on the mechanical properties and microstructure of carbon-fiber-reinforced coral concrete (CFRCC) by means of mechanical experiments, X-ray diffractometry, digital microscopy and scanning electron microscopy, finding that the addition of carbon fibers was able to improve the compressive strength and splitting tensile strength of concrete. Methods for improving the mechanical properties of SCAC by incorporating additives are also receiving attention [23]. Cheng et al. [24] studied the effects of the addition of waste ash (FA), blast furnace slag (BFS) and metakaolin (MK) on the mechanical properties, drying shrinkage, carbonation and chloride ion permeability of coral sand concrete (CSC), and compared the results with ordinary Portland cement (OPC) and natural aggregate concrete (NAC), concluding that the compressive strength of CSC was slightly lower, but possessed better chloride ion permeability. Islands possess abundant reef limestone resources, and the main component of reef limestone is CaCO_3_ [25], which is the main raw material for the production of limestone powder. Studies have shown that limestone powder has a positive effect on improving the mechanical properties of concrete [26,27,28,29]. However, there is still a lack of reports studying the addition of limestone powder to SCAC. Whether limestone powder can also improve the mechanical properties of SCAC and the effect of limestone powder content on the mechanical properties of SCAC deserves further exploration.

In addition, the problem of how to improve the dynamic mechanical properties of SCAC when presented with the risks of dynamic loading, resulting from phenomena such as earthquakes or explosions, have also drawn the attention of researchers. Ma et al. [30] conducted dynamic impact loading tests using a split Hopkinson pressure bar (SHPB) system with a diameter of 100 mm, and the effects of strain rate on the uniaxial compressive strength, energy dissipation, fractal dimension, and failure morphology of SCAC were studied. Ma et al. [20,31] proposed sisal-fiber-reinforced CASC (SFCASC) with a compressive strength of 77.3 MPa. Dynamic mechanical experiments were performed on the SFCASC by SHPB, and the SFCASC was found to exhibit an obvious strain rate effect. It can be found from the above research that a number of researchers have used the SHPB test system to investigate the dynamic mechanical properties of SCAC. In fact, the SHPB experimental technology has been widely used in the study of the dynamic mechanical properties of concrete materials [32] due to its good performance when testing dynamic mechanical properties at strain rates in the range of 10^1^~10^4^ s^−1^ [33]. Moreover, with the development of SHPB technology, researchers have combined high-speed photography technology [34], coupled static-dynamic loading [35], Digital Image Correlation (DIC) [36] and other technologies with the traditional SHPB experimental system, greatly expanding the use scenarios of SHPB test systems. In addition, DIC technology has attracted the attention of researchers because of the advantage in terms of the strain field of the measured specimens being able to be measured directly using non-contact methods during the process of performing mechanical experiments [37].

Therefore, in this paper, in order to explore the influence of limestone powder content on the dynamic and static mechanical properties of SCAC, SCAC specimens with different limestone powder contents were processed. Quasi-static and dynamic mechanical experiments carried out on SCAC with different limestone powder contents using RMT, SHPB and high-speed camera. The properties tested in the dynamic and static experiments included static compressive strength, static tensile strength, dynamic tensile strength, DIF, dynamic strain field and failure pattern. The influence of limestone powder content on the dynamic and static mechanical properties of SCAC was studied.

## 2. Materials and Methods

### 2.1. Raw Materials

**Figure 1:**
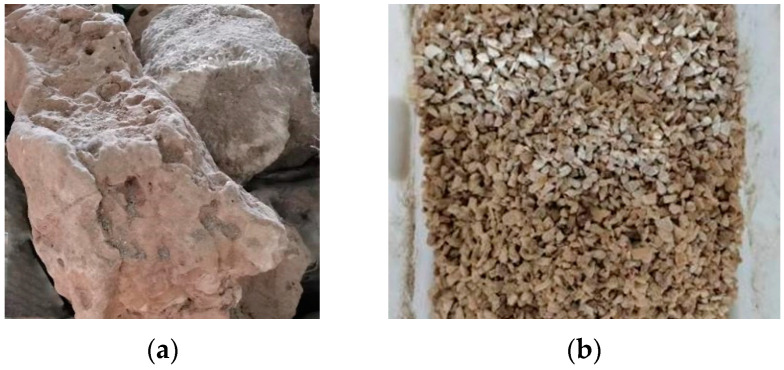
Reef limestone aggregate. (**a**) Original reef limestone; (**b**) reef limestone aggregate.

**Figure 2:**
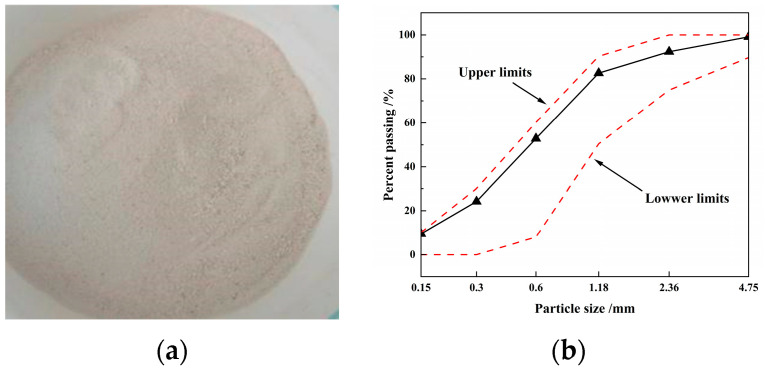
Coral sand from reef: (**a**) image of the coral sand from the reef; (**b**) particle size distribution of the coral sand.

**Figure 3:**
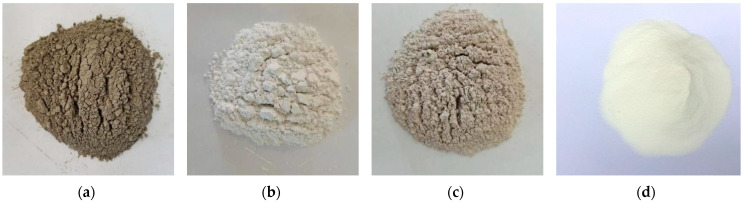
Image of cementitious material and polycarboxylate superplasticizer: (**a**) cement; (**b**) limestone powder; (**c**) slag powder; (**d**) polycarboxylate superplasticizer.

Reef limestone (Figure 1) and coral sand (Figure 2) collected directly from the reef were selected for the coarse aggregate and fine aggregate, respectively, of the SCAC. The basic physical properties of reef limestone and coral sand shown in Table 1 were tested in accordance with the Chinese standard GB/T 14685-2022 [38]. Figure 2b shows the particle size distribution of the coral sand tested using the method described in the literature [39]. The binding materials used in the experiment mainly include cement, limestone powder and slag powder (Figure 3). The cement was P.O52.5 Portland cement, produced by Zhuchengyangchun Cement Co., Ltd. (Weifang, China), which satisfied the requirements of the Chinese standard GB175-2007 [40]. The performance of polycarboxylate superplasticizer produced by Hongxiang Construction Admixture Factory in Laiyang City, Shandong Province was able to satisfy the requirements of the Chinese standard GB/8076-2008 [41]. The artificial seawater was configured in accordance with the literature [12], rather than using fresh water for the formulation of the SCAC.

### 2.2. Mix Proportion and Sample Preparation

**Figure 4:**
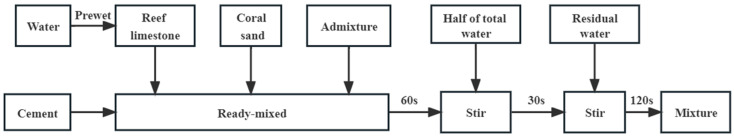
Production process of the SCAC specimens.

The mix proportions of SCAC in this study, shown in Table 2, were calculated using Equation (1), in accordance with the Chinese standard JGJ 51-2002 [42]. The specimens that were subjected to quasi-static mechanical testing and dynamic mechanical testing were Φ50 mm × 100 mm and Φ65 mm × 35 mm cylindrical specimens, with 3 molded specimens in each group. The manufacturing process of the concrete specimens is shown in Figure 4. The concrete was poured by vibration to ensure the uniformity of coarse aggregate in concrete. All SCAC specimens underwent 28 days of curing under the same conditions, following the curing method described in the literature [43]. In order to ensure the smoothness of the surface of the specimen, the upper and lower surfaces of the specimen were polished using a grinding machine after maintenance.

(1) $$f_{cu,o}\geq f_{cu,k}+1.645\sigma$$

where $f_{cu,o}$ and $f_{cu,k}$ represent the trial strength of the lightweight aggregate concrete and the standard cube compressive strength value of the lightweight aggregate concrete, respectively; σ represents the standard deviation of the strength of the lightweight aggregate concrete.

### 2.3. Static Compressive and Tensile Strength Experiment

**Figure 5:**
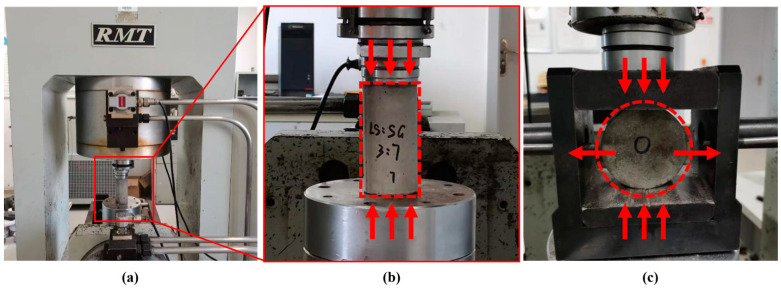
Quasi-static mechanical testing: (**a**) RMT-150B testing system; (**b**) quasi-static compressive testing; (**c**) quasi-static tensile testing.

Quasi-static compressive and tensile testing of the SCAC was carried out using an RMT-150B experimental machine (Figure 5, Wuhan Institute of Geotechnical Mechanics, Wuhan, China). The RMT-150B rock mechanics test system consists of four parts: the host, the hydraulic system, the servo control system, and the computer control and processing system [44]. The Brazilian disc method [45] was applied to perform tensile strength testing of the specimen by transferring stress to the specimen in the tensile and compressive directions.

### 2.4. Dynamic Mechanical Properties Experiment

**Figure 6:**
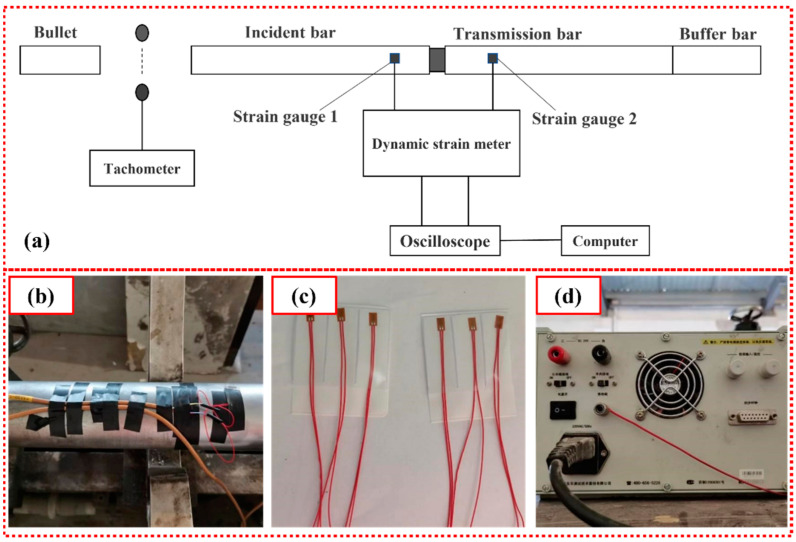
SHPB experimental system: (**a**) schematic diagram of SHPB experimental device; (**b**) bonding method of strain gauge; (**c**) strain gauge; (**d**) dynamic strain meter.

The Split Hopkinson Pressure Bar (SHPB) is a test system that can be used to effectively test the dynamic mechanical properties of materials under strain rates in the range 10^1^~10^4^ s^−1^, and has been widely used for testing the dynamic mechanical properties of rock, concrete and other geotechnical engineering materials [46,47]. The SHPB consists of a launcher, a bullet, an incident bar, a transmission bar, a buffer bar, a strain gauge attached to the bar, a speed test system, a dynamic strain meter, and an analysis system. A schematic diagram for the SHPB test device is presented in Figure 6.

**Figure 7:**
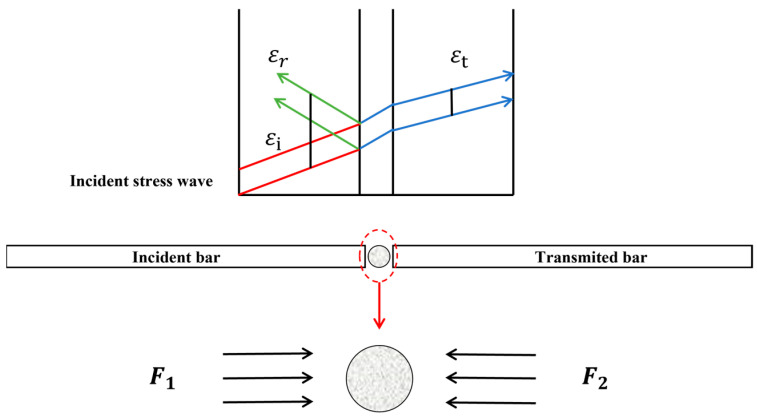
Stress wave transmission during the SHPB test. *F*_1_, *F*_2_ represent the stress direction of both sides of the specimen, respectively.

The transmission of the stress wave in the SHPB test is shown in Figure 7. During the SHPB test, under the impetus of high-pressure gas, the bullet leaves the launcher to impact the incident bar, resulting in an incident stress wave. When the incident stress wave propagates between the incident bar and the specimen, the specimen is compressed in the direction along the bar. Because of the difference in the wave impedance between the bar and the specimen, some of the incident stress waves will become reflected stress waves, while the others will become transmitted stress waves when they penetrate the transmission bar. These 3 waves are measured by resistance strain gauges attached to the incident bar and the transmission bar, respectively. Finally, the electrical signal collected by the strain gauge is output by the computer acquisition system, and the impact data of the material are finally obtained.

Two assumptions should be satisfied when analyzing the SHPB test results [48]: (1) One-dimensional stress wave assumption: it must be ensured that the wavelength of the propagating stress wave is much larger than the diameter of the compression bar and that the compression bar is an elastic bar, while the compression bar can only undergo axial deformation, and the stress wave can only propagate along the axial direction; (2) Stress uniformity assumption: the test must ensure that the specimen is small enough to ensure that the stress and strain state inside the specimen is evenly distributed during the loading process. The formula used for data processing can be derived on the basis of these two assumptions (Equation (2)) [30], and the dynamic tensile stress of the specimen can be obtained based on the data obtained from the SHPB experiment, in line with the principle of the dynamic Brazilian disc splitting experiment (Equation (3)) [49].

(2) $$\left\{\begin{array}{l} \varepsilon_{s}\left(t\right)=\frac{c_{0}}{D}\int_{0}^{t}\left[\varepsilon_{i}\left(t\right)-\varepsilon_{r}\left(t\right)-\varepsilon_{t}\left(t\right)\right]dt\\ \sigma_{s}\left(t\right)=\frac{EA}{\pi DB}\left[\varepsilon_{i}\left(t\right)+\varepsilon_{r}\left(t\right)+\varepsilon_{t}\left(t\right)\right]\\ {\dot{\varepsilon }}_{t}\left(t\right)=\frac{c_{0}}{D}\left[\varepsilon_{i}\left(t\right)-\varepsilon_{r}\left(t\right)-\varepsilon_{t}\left(t\right)\right] \end{array}\right.,$$

where *A* and *E* represent the cross-sectional area and elastic modulus of the bar, respectively; D and B represent the diameter and thickness of the specimen, respectively; $\varepsilon_{i}\left(t\right)$, $\varepsilon_{r}\left(t\right)$ and $\varepsilon_{t}\left(t\right)$ represent incident strain, reflected strain and transmitted strain, respectively; $\varepsilon_{s}\left(t\right)$, $\sigma_{s}\left(t\right)$ and ${\dot{\varepsilon }}_{t}\left(t\right)$ represent strain, stress and strain rate, respectively.

(3) $$\sigma_{t}=\frac{2P}{\pi DB},$$

### 2.5. Digital Image Correlation Method

**Figure 8:**
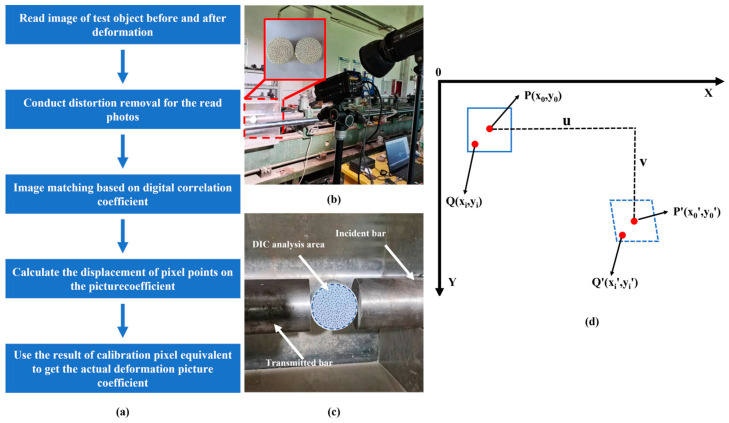
Digital image correlation method. (**a**) Flowchart of the digital image correlation method; (**b**) DIC experimental apparatus; (**c**) DIC analysis area; (**d**) relationship between the reference sub-region and the deformation sub-region before and after deformation.

The DIC method (Figure 8) can be used to analyze the information at a specific point based on the change in the shape and position of the speckles on the surface of the specimen when applying force to the object [50]. In order to ensure that the image conditions satisfy the recognition requirements, the specimen needs to be sprayed in a ‘scattered spot’ manner (Figure 8b). The basic principle of the digital image correlation method (Figure 8d) is to select a square image sub-region, where the center of the sub-region is the pixel point. 

## 3. Results and Analysis

### 3.1. Static Test Results and Analysis

**Figure 9:**
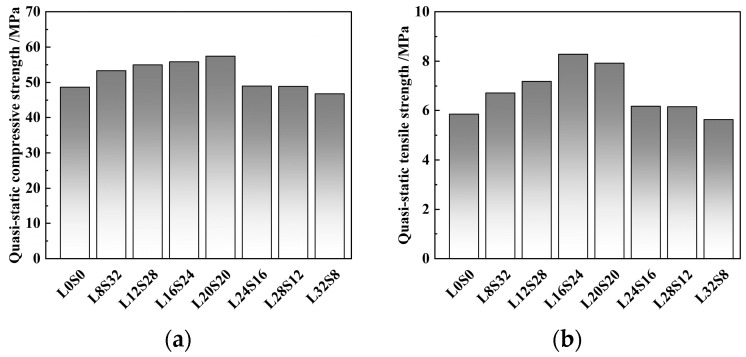
Compressive strength and tensile strength of concrete specimens with different contents: (**a**) compressive strength; (**b**) tensile strength.

**Figure 10:**
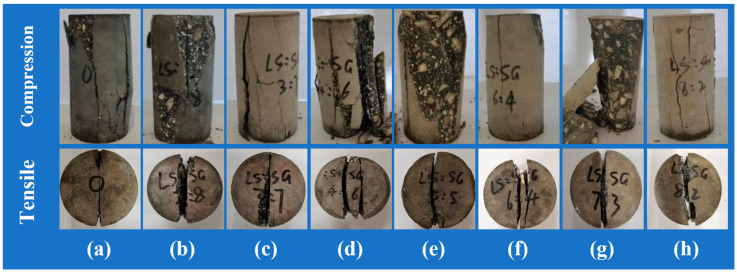
The typical failure morphology of SCAC in quasi-static compression and tensile tests: (**a**) L0S0; (**b**) L8S32; (**c**) L12S28; (**d**) L16S24; (**e**) L20S20; (**f**) L24S16; (**g**) L28S12; (**h**) L32S8.

The quasi-static compressive strength and quasi-static tensile strength of SCAC with different mixing ratios of limestone powder and slag powder were statistically analyzed, and the results were as shown in Figure 9. Figure 10 shows the failure morphology of SCAC under static compressive and static tensile tests with different dosage ratios of limestone powder and slag powder.

It can be seen from Figure 9a that the addition of limestone powder and slag powder influenced the static compressive strength of SCAC, and the ratio of limestone powder and slag powder had a significant effect on the quasi-static compressive strength of coral sand concrete. Compared with SCAC (L0S0) without the addition limestone powder and slag powder, the static compressive strength of SCAC with 8~20% limestone powder and 20~32% slag powder increased with increasing dosage of limestone powder and decreasing dosage of slag powder. According to the different dosage ratios of limestone powder and slag powder (2:8, 3:7, 4:6, 5:5), the quasi-static compressive strength of SCAC increased by 9.53%, 12.94%, 14.75% and 17.97%, respectively, reaching a maximum value of 57.4 MPa when the limestone powder dosage was 20% and the slag powder dosage was 20%. However, when the limestone powder content was greater than 20%, the quasi-static compressive strength of SCAC with limestone powder and slag powder ended its downward trend with increasing limestone powder content, and when the limestone powder content was 32% and slag powder content was 8%, the addition of limestone powder and slag powder caused the quasi-static compressive strength of SCAC to decrease by 3.48%. By comparing Figure 9a,b, it can be found that the limestone powder content and slag powder also affected the quasi-static tensile strength of the SCAC, and it can be observed that the strength first increased and then decreased with increasing limestone powder dosage.

It can be observed from Figure 10 that the failure morphology of SCAC specimens under quasi-static compression loading is dominated by shear failure and accompanied by intermediate tensile failure. The expansion and penetration of cracks are the main factors that led to the failure of the SCAC specimens. Different of limestone powder and slag powder contents affected the failure morphology and post-failure morphology of SCAC. With increasing limestone powder content, the axial cracks of SCAC gradually decreased, the inclined cracks gradually increased, and the failure morphology gradually developed from complete crushing splitting failure to oblique shear failure with the tensile effect. When limestone powder content was 20% and slag powder content was 20% (L20S20, Figure 10e), the SCAC underwent typical oblique shear failure. The specimen broke into two main fragments along the shear surface, and the specimen after failure still had a certain bearing capacity. However, when the limestone powder content exceeded 20%, the failure morphology of SCAC began to develop into the form of tensile failure. The expansion of multiple parallel axial cracks led to a decrease in the bearing capacity of the fragments after the failure of the specimens, and the degree of fragmentation increased. After the static tensile test, SCAC underwent typical radial splitting failure, and the fracture end surface were relatively flat. Different limestone powder and slag powder contents affected the static tensile failure form and post-failure form of the SCAC. With increasing limestone powder content, more than a fracture surface began to appear. The increase in limestone powder content and the decrease in slag content affected the crack resistance of the SCAC.

Based on the above experimental phenomena described above, it can be seen that limestone powder and slag powder contents were between 8~20% and 20~32%, respectively, facilitated the improvement of quasi-static compressive strength and tensile strength of the coral concrete. Studies have shown that the interaction between sulfate and Ca(OH)_2_ affects the strength of concrete during the hydration process of the concrete, and the addition of limestone powder and slag powder to concrete can effectively alleviate the effect of sulfate and Ca(OH)_2_ [29]. In the early stage of cement hydration, CaCO_3_ particles in limestone powder play the role of the crystal nucleus in the Ca(OH)_2_ and C-S-H produced by cement hydration, accelerating the hydration of clinker minerals such as C_3_S [51], thus effectively improving the early strength of concrete. In the later stage of cement hydration, higher ratios of the reaction of C_3_S might relatively decrease the content of C_2_S, which may be the responsible for subsequent strength development [52].

### 3.2. Dynamic Mechanical Testing Results and Analysis

#### 3.2.1. Typical Stress Waveform

**Figure 11:**
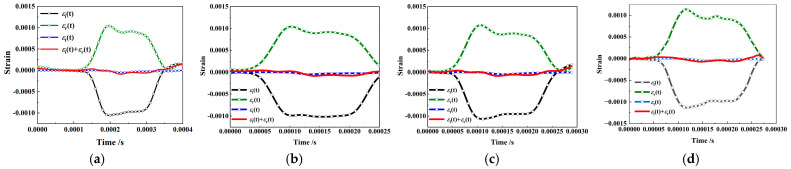
Typical dynamic stress equilibrium verification records of SCAC specimens in SHPB experiment: (**a**) LS:SG = 2:8, 169.32 s^−1^, (**b**) LS:SG = 4:6, 171.1 s^−1^, (**c**) LS:SG = 5:5, 181.258 s^−1^, (**d**) LS:SG = 7:3, 167.74 s^−1^.

Figure 11 shows the typical dynamic stress equilibrium verification of SCAC specimens with various ratios of limestone powder and slag powder (LS:SG = 2:8, 4:6, 5:5, 8:2) in the SHPB experiment. The validity of the experimental data was determined on the basis of the dynamic stress balance in the SHPB experiment according to the method described in the literature [53]. It can be observed from Figure 11 that there was a similar trend between “*ε*_t_(t)” and “*ε*_i_(t) + *ε*_r_(t)” in the SHPB experiment on the SCAC, meaning that the dynamic stress equilibrium conditions were basically satisfied. The satisfaction of dynamic stress equilibrium conditions provided favorable evidence for the constant strain rate loading and verified the validity of the experimental results.

#### 3.2.2. Stress–Strain Curve

**Figure 12:**
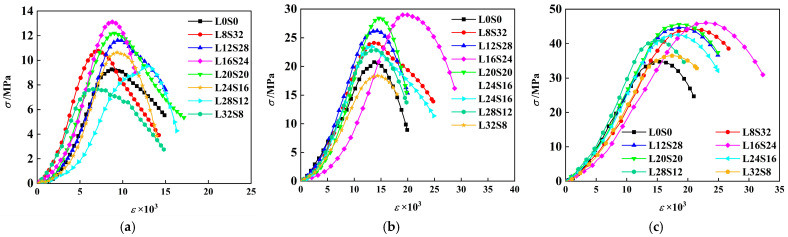
Stress–strain curves under different strain rates of SCAC: (**a**) low degree of strain rate (153.85~171.1 s^−1^); (**b**) medium degree of strain rate (301.1~343.17 s^−1^); (**c**) high degree of strain rate (538.33~558.4 s^−1^).

The dynamic tensile stress–strain curves with different limestone powder and slag powder contents under different strain rates were obtained by processing the original waveform data, as shown in Figure 12. It should be noted that the “low”, “medium” and “high” strain rate levels mentioned here are only used to facilitate their naming, and do not express the same concepts as “low strain rate”, “medium strain rate” and “high strain rate”, in the strict sense [33].

It can be observed that the dynamic tensile stress–strain curve of SCAC has similar characteristics to those of other concrete materials in the SHPB experiment, showing four stages: a compaction stage, an elastic stage, a crack development stage, and a failure stage.

- Compaction stage (I): because there are fine cracks inside the concrete that close under the action of external forces, the curve shows a slow upward trend of strain hardening.
- Elastic stage (II): the specimen undergoes elastic-like deformation, and the curve grows in a nearly linear manner.
- Crack generation and propagation stage (III): microcracks begin to appear inside the specimen, and as the stress increases, the concrete specimen is destroyed. The cracks inside the concrete form rapidly, the density gradually increases, the stress reaches the maximum value, and the concrete also reaches its maximum bearing capacity.
- Fracture and failure stage (IV): the strain continues to increase, while the bearing capacity of the concrete decreases. At this stage, the micro-cracks of the concrete gradually penetrate until the specimen is complete destroyed.

#### 3.2.3. Strain Rate Effect

**Figure 13:**
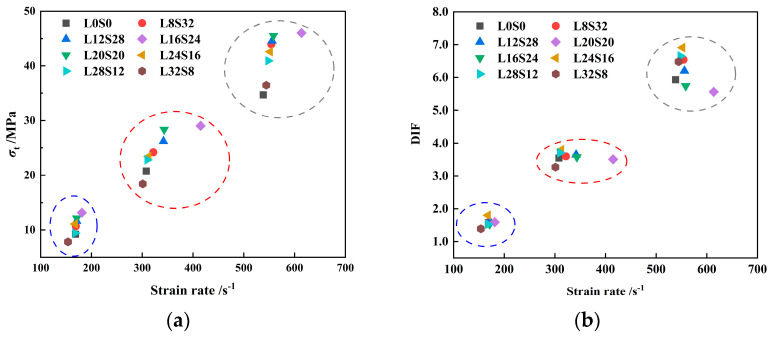
The peak stress and *DIF* of SCAC in SHPB experiment: (**a**) peak stress; (**b**) *DIF*.

In addition to the characteristics of the stress–strain curve, SCAC also showed an obvious strain rate effect, similar to other concrete materials [36,54,55]. It can be seen from Figure 13a that the tensile stress–strain curve of SCAC indicated an increase in peak stress with increasing strain rate level.

Dynamic Increase Factor (DIF) [56] (Equation (4)), a common index, can be used to investigate the sensitivity of materials to strain rate. The peak stress and DIF of the SCAC in the experiment are shown in Figure 13b.

(4) $$DIF=\frac{\sigma_{t}}{\sigma_{s}},$$

where $\sigma_{t}$ and $\sigma_{s}$ represent the dynamic tensile strength and static tensile strength, respectively, of SCAC.

**Figure 14:**
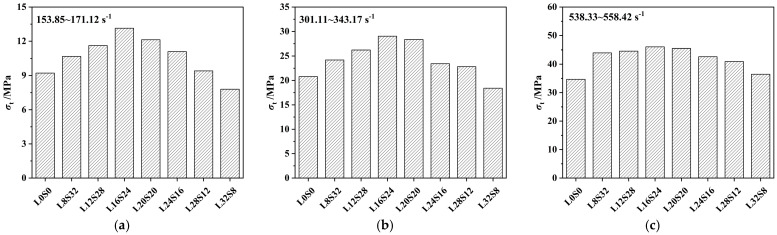
The dynamic tensile strength of SCAC with different limestone powder and slag powder contents under different strain conditions: (**a**) strain rate = 171.12~153.85 s^−1^; (**b**) strain rate = 301.11~343.17 s^−1^; (**c**) strain rate = 538.33~558.42 s^−1^.

**Figure 15:**
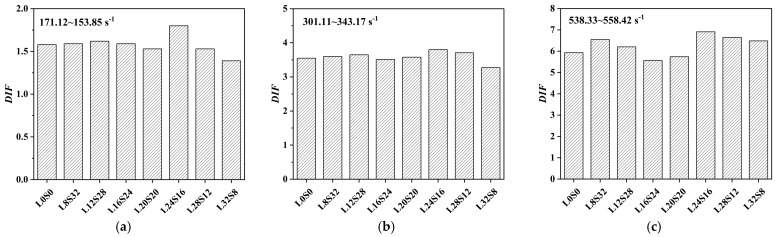
The *DIF* of SCAC with different limestone powder content and slag powder under different strain rate conditions: (**a**) strain rate = 171.12~153.85 s^−1^; (**b**) strain rate = 301.11~343.17 s^−1^; (**c**) strain rate = 538.33~558.42 s^−1^.

It can be observed from Figure 13a that the dynamic tensile strength of SCAC mainly fluctuates in the range of 7.8 MPa to 46.01 MPa. The dynamic tensile strength of SCAC with the same ratio increased with increasing strain rate grade. In addition, with increasing strain rate, the *DIF* of the SCAC under different ratio conditions also showed an increasing trend, with the *DIF* of the SCAC varying from 1.39 to 6.91. In order to better observe the effect of varying limestone powder and slag powder contents on the dynamic tensile strength and *DIF* of SCAC, the dynamic tensile strength and *DIF* of SCAC with different strain rates and different contents were determined, and the results are statistically shown in Figure 14 and Figure 15.

**Figure 16:**
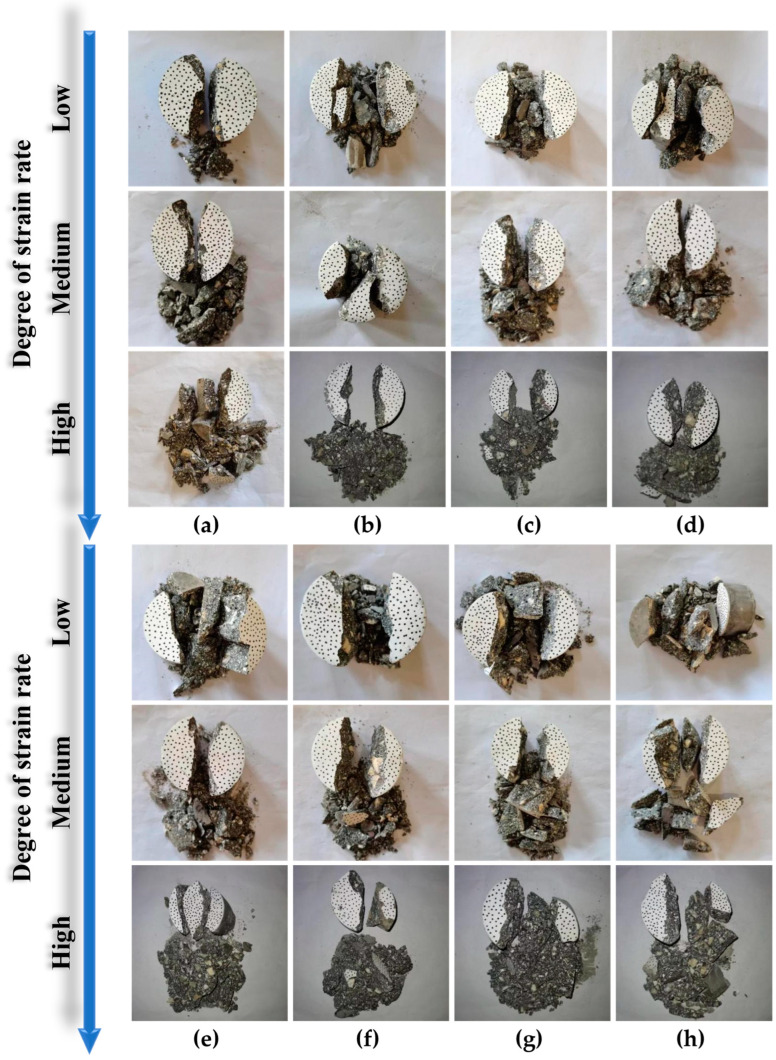
The failure pattern of SCAC during the tensile SHPB test. (**a**) L0S0; (**b**) L8S32; (**c**) L12S28; (**d**) L16S24; (**e**) L20S20; (**f**) L24S16; (**g**) L28S12; (**h**) L32S8.

It can be observed from Figure 14 and Figure 15 that under the same strain rate level, the dynamic tensile strength of SCAC increased at the beginning and then decreased with increasing limestone powder content, reaching its maximum value when the limestone powder content was 16% and the slag powder content was 24%. However, the addition of limestone powder did not completely guarantee an improvement in the dynamic tensile strength of SCAC. At all strain rate grades, the dynamic tensile strength of SCAC decreased under the condition of 32% limestone powder content and 8% slag powder content, and this attenuation effect was more obvious at low strain rate levels (171.12~153.85 s^−1^). At the same strain rate level, the *DIF* of SCAC did not show the same trend as dynamic tensile strength. With increasing limestone powder content, the *DIF* of SCAC exhibited a fluctuation phenomenon around a specific value, which was 1.57, 3.58 and 6.25 at different strain rate levels. In addition, at all strain rate levels, the *DIF* of SCAC reached its maximum value under the condition of 24% limestone powder content and 16% slag powder content, and this maximum value increased with increasing strain rate level. These rules seem to correspond to the failure pattern of SCAC in the SHPB experiment (Figure 16). It can be seen from Figure 16 that under dynamic tensile conditions, although the SCAC specimens with typical splitting failure did not break completely, the failure morphology of SCAC specimens showed a trend in which the particle size of the broken slag decreased, with the amount of broken slag increasing, as well as breaking more thoroughly, with increasing strain rate grade.

As mentioned above, the addition of limestone powder to SCAC affects the strength of the SCAC by affecting the C-S-H in the concrete. The addition of limestone powder affects the quasi-static strength of the SCAC. However, under dynamic load with high strength and a short action time, this effect was not obvious. Although C-S-H can fill the pores and micro-cracks in SCAC and reduce the porosity of concrete, the bonding effect of the limestone powder was shown macroscopically. However, once the limestone powder content had exceeded a certain threshold, the resulting C-S-H was not only not able to continuously fill the pores and microcracks, it also had a tendency to increase the porosity [57]. This can be proved by performing both quasi-static and dynamic mechanical experiments on SCAC. In addition, under the action of dynamic load, the rapid tensile effect caused by dynamic load input in a short time was much higher than the enhancement of the bonding ability of SCAC caused by C-S-H. Therefore, in the dynamic splitting test, the dynamic tensile strength of SCAC with different limestone powder contents at the same strain rate level showed obvious differences, and these differences increased with increasing strain rate grade.

### 3.3. Failure Process and Strain Field of SCAC in the SHPB Experiment

**Figure 17:**
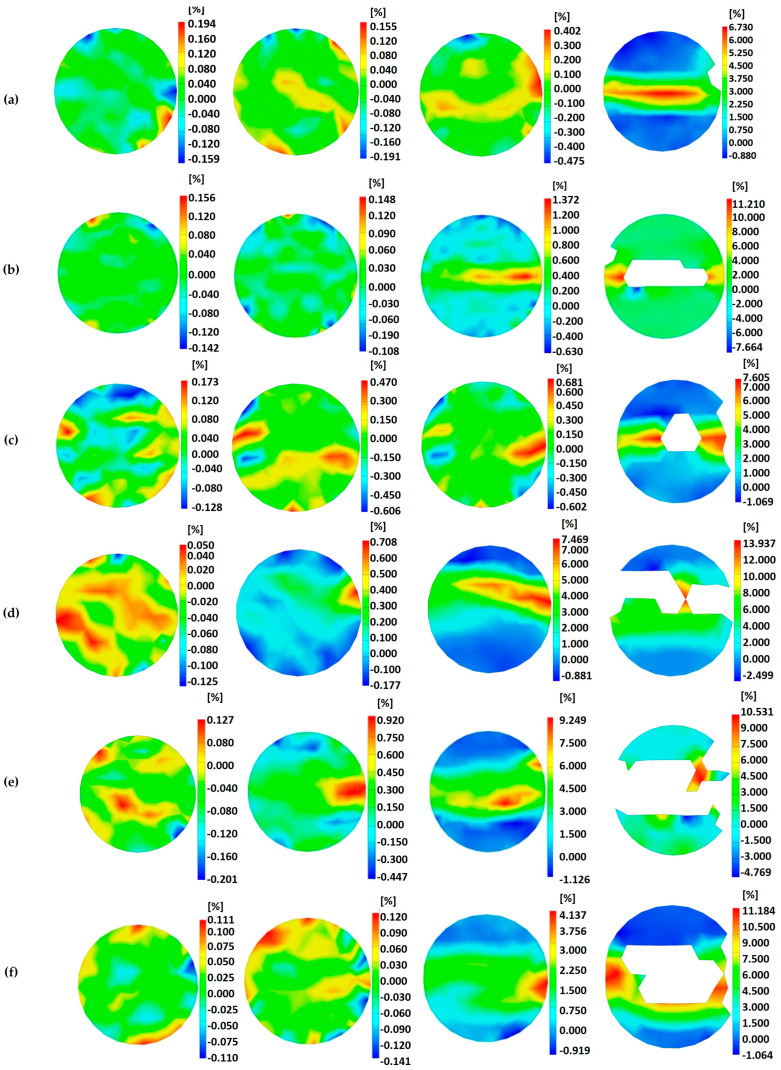

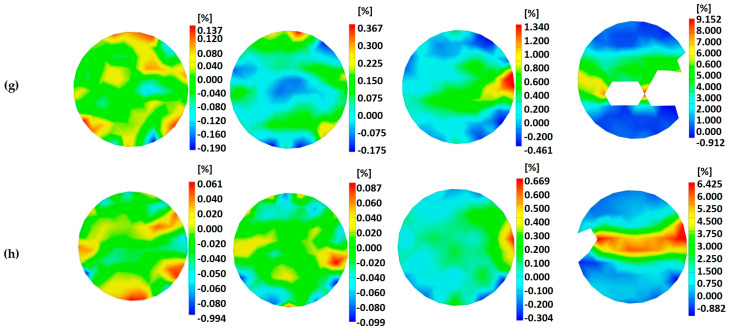
Evolution of vertical strain field of SCAC with different limestone powder contents under strain rate= 153.85 s^−1^-171.11 s^−1^: (**a**) L0S0; (**b**) L8S32; (**c**) L12S28; (**d**) L16S24; (**e**) L20S20; (**f**) L24S16; (**g**) L28S12; (**h**) L32S8.

**Figure 18:**
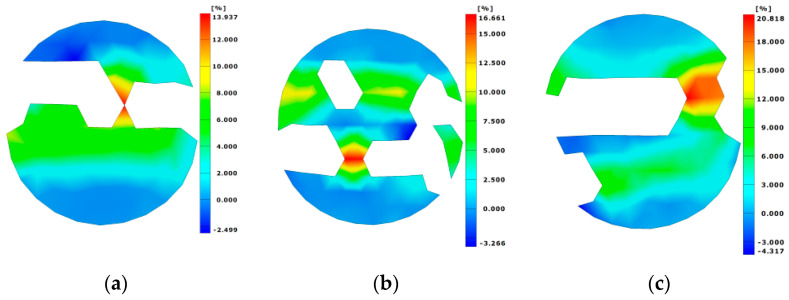
The distribution of the strain field in the failure stage of SCAC under different strain rate grades. (**a**)Low; (**b**) Medium; (**c**) High.

The failure process of SCAC in the SHPB experiment was observed by means of high-speed camera technology, and the strain field change in the SCAC during the dynamic tensile process was observed by DIC technology. Figure 17 shows the strain field distribution of the SCAC with different limestone powder contents along the Y direction under typical strain rate conditions (301.1~343.17 s^−1^). The upward stress is positive, and the downward stress is negative. In addition, in order to better compare the strain field distribution of the SCAC under impact loading under different strain rate conditions, in Figure 18, the strain field of the SCAC in the failure stage was quantified under different strain rate conditions.

It can be seen from Figure 17 that the development process of the strain field of the SCAC with different limestone powder contents possessed a unified trend. From left to right are the images of the initial loading stage, the crack generation stage, the crack propagation stage, and the complete failure stage of the specimen. In the early stage of impact loading, the deformation and strain accumulation of the SCAC first appeared in the area of contact with the bar. As the impact load continued to act on the SCAC specimen, symmetrical cracks began to appear along the symmetrical direction in the SCAC strain field, symmetrically distributed from the midline to the edge of the specimen, which began to expand.

In the later stage of impact loading, the propagation and interconnection of cracks caused penetrating cracks to appear, followed by the failure of the specimens. The above phenomenon is consistent with the results of the numerical calculation of dynamic splitting in SCAC carried out by Ma et al. [31] using LS-DYNA software, where it was found that the failure of the SCAC specimen showed that the external failure of the specimen was greater than the internal failure, and the central failure was greater than the edge failure. In addition, when comparing the strain field of the SCAC in the failure stage under different strain rate conditions (Figure 18), the maximum strain of SCAC showed an increasing trend with the increase in strain rate level, thus demonstrating obvious strain rate sensitivity. However, at the same strain rate grade, the change trend of the maximum strain value of SCAC with different limestone powder contents was not consistent with the change trend for stress.

## 4. Conclusions

1. The quasi-static mechanical properties of SCAC are closely related to the content of limestone powder. When the limestone powder content was between 8% and 20%, an improvement in the quasi-static compressive and tensile strength of the SCAC was achieved, and the improvement in the quasi-static compressive strength was the best when the limestone powder content was 20%. The improvement in the quasi-static tensile strength was the best when the limestone powder content was 16%.
2. The dynamic tensile strength of SCAC demonstrated an obvious strain rate effect under dynamic load, and the dynamic tensile strength and DIF of SCAC increased with increasing strain rate grade; with increasing limestone powder content, the dynamic tensile strength of SCAC showed first an increasing trend and then a decreasing trend, reaching its maximum value when the limestone powder content was 16%.
3. Under dynamic tensile loading, the failure of SCAC was caused by the development, connection and penetration of microcracks, and the failure position tended to expand from the edge to the center. The maximum strain value of SCAC with the same limestone powder content increased with increasing strain rate grade. Under the same strain rate, the change trend of the maximum strain value of SCAC with different limestone powder contents was not consistent with the change trend of stress, but fluctuated around a certain value.

## Figures and Tables

**Table 1:** The basic physical properties of coarse aggregate and fine aggregate.

Aggregate	Apparent Density (kg/m^3^)	Bulk Density (kg/m^3^)	Porosity (%)	One Hour Water Absorption (%)
Coarse	1471	828	43.67	18.20
Fine	1864.2	1209	36.75	6.26

**Table 2:** Mix proportions of the coral aggregate concrete.

Number	(LS:SG)	Cement	Limestone Powder	Slag Powder	Reef Limestone	Coral Sand	Water	Slushing Agent
L0S0	-	550	0	0	680	1020	165	8.25
L8S32	2:8	330	44	176	680	1020	165	8.25
L12S28	3:7	330	66	154	680	1020	165	8.25
L16S24	4:6	330	88	132	680	1020	165	8.25
L20S20	5:5	330	110	110	680	1020	165	8.25
L24S16	6:4	330	132	88	680	1020	165	8.25
L28S12	7:3	330	154	66	680	1020	165	8.25
L32S8	8:2	330	176	44	680	1020	165	8.25

Note: “L” represents limestone powder, while “S” represents slag powder, and the number after the letter indicates the amount of slag admixture. For example, “L8S32” denotes SCAC with 8% limestone powder and 32% slag powder.

## Data Availability

Not applicable.
